# Sourdough Fermentation Improves the Antioxidant, Antihypertensive, and Anti-Inflammatory Properties of *Triticum dicoccum*

**DOI:** 10.3390/ijms24076283

**Published:** 2023-03-27

**Authors:** Morena Gabriele, Nafiou Arouna, Július Árvay, Vincenzo Longo, Laura Pucci

**Affiliations:** 1 Italian National Research Council, Institute of Agricultural Biology and Biotechnology, Via Moruzzi 1, 56124 Pisa, Italy; vincenzo.longo@ibba.cnr.it (V.L.); laura.pucci@ibba.cnr.it (L.P.); 2 Department of Agricultural Sciences, University of Naples Federico II, Via Università 100, 80055 Naples, Italy; nafiou.arounaa@gmail.com; 3 Institute of Food Sciences, Faculty of Biotechnology and Food Sciences, Slovak University of Agriculture, 949 76 Nitra, Slovakia; julius.arvay@uniag.sk

**Keywords:** *Triticum dicoccum*, sourdough fermentation, bioactive compounds, antioxidant activity, HPLC-DAD, CAA-RBC, hemolysis test, ACE-inhibitory activity, HT-29, inflammation

## Abstract

The fermentation process has been widely used to improve plant-based foods’ nutritional and nutraceutical properties. This study aimed to investigate and compare the impact of sourdough fermentation on the bioactive content and profile, antioxidant and antihypertensive activities, as well as the anti-inflammatory properties of fermented (FS) and non-fermented (NFS) flour from Tuscan *Triticum dicoccum* wheat (spelt) on tumor necrosis factor-alpha (TNF-α)-inflamed human intestinal epithelial cells (HT-29). FS showed significantly higher total phenolic and flavonoid content, in vitro and ex vivo antioxidant activities, and ACE-inhibitory activities than NFS. Gallic acid was identified by HPLC-DAD as the most representative polyphenol, followed by rutin, trans-ferulic acid, iso-quercitrin, and quercetin, in the fermented spelt sample. Instead, rutin and gallic acid were identified as the predominant compounds in the non-fermented ones. Moreover, FS exhibited a better protective effect on inflamed HT-29 cells by significantly counteracting the TNFα-induced alterations, lowering the expression of IL-8, COX-2, and ICAM-1 inflammatory mediator while enhancing antioxidant enzyme HO-1 gene expression. In conclusion, sourdough fermentation positively affected the nutraceutical and functional properties of spelt, which may represent a valuable ingredient for the formulation of functional foods and a key product for managing hypertension and inflammatory intestinal diseases.

## 1. Introduction

In recent years, fermented foods and ancient cereal-based products have gained considerable interest in Western countries due to their health benefits. Fermentation has been reported to improve the nutritional and nutraceutical properties of several plant based-foods [1]. In contrast, ancient cereals have recently been recognized as sustainable healthy foods because of their high content of bioactive components and low negative environmental impact [2,3]. Fermentation results in chemical composition changes, increases functional compounds, and further improves the health-promoting effects of derived products [4]. However, not all aspects of sourdough fermentation’s effects on *Triticum dicoccum* wheat functional properties have been thoroughly investigated.

*Triticum dicoccum*, also known as spelt, emmer wheat, or farro in Italy, is one of the oldest cereals used by men, whose cultivation dates to around 7000 BC, that is gaining attention in recent years for its supposed health-promoting effect and sustainable production. It has been reported to contain a high concentration of proteins, dietary fiber, and antioxidant components. In addition, *T. dicoccum* has a lower starch digestibility than other species of wheat, being more suitable for diabetic patients [5,6]. Thorup et al. [7] reported that *T. dicoccum* significantly decreased glycemic response in type 2 diabetes animal models compared to a modern wheat diet. Moreover, a diet based on *T. dicoccum* significantly reduced the total cholesterol, triglycerides, and LDL-cholesterol of type 2 diabetic patients compared to the modern wheat diet [8]. Instead, Christopher et al. [9] observed a higher phenolic content, as well as higher antioxidant and antihyperglycemic activities, in *T. dicoccum* compared to two commercial wheat cultivars. This evidence highlighted the importance and health benefits of *T. dicoccum*.

Sourdough fermentation is an ancient practice used to leaven the dough. It is well-documented that sourdough fermentation improves foods’ shelf life, digestibility, and protein content. It reduces antinutritional factors such as phytates, tannins, trypsin inhibitors, and fermentable oligo-, di-, monosaccharides, and polyols (i.e., FODMAPs), which aggravate irritable bowel syndrome symptoms. In addition, sourdough fermentation increases the presence of bioactive components with various functions, including antioxidant, antihypertensive, and anti-inflammatory activities [10,11]. Nowadays, for their limited side effects and valuable bio-functional properties, natural products and food-derived compounds represent promising tools for the management and/or treatment of hypertension and intestinal inflammation [12,13,14].

The impacts of sourdough fermentation on the nutraceutical properties of some ancient cereals have been recently summarized by Șerban et al. [15]. However, only a few studies have investigated the effects of sourdough fermentation on the bioactive compounds and bio-functional properties of *T. dicoccum*. Among others, Colosimo et al. [16] reported higher concentrations of amino acids, total polyphenols, and antioxidant activities in sourdough-fermented *T. dicoccum* than the non-fermented one. In a similar experiment, the effects of sourdough fermentation with lactic acid bacteria and chemically acidified doughs on *T. dicoccum* flour were compared; authors observed a considerable increase in the antioxidant peptides and radical-scavenging activity in sourdough fermented *T. dicoccum* to the chemically fermented one [17].

This study aimed to investigate and compare the impact of sourdough fermentation on the bioactive content and composition, antioxidant and antihypertensive activities, as well as the anti-inflammatory properties of fermented (FS) and non-fermented (NFS) flour from Tuscan *T. dicoccum* on tumor necrosis factor-alpha (TNF-α)-inflamed human intestinal epithelial cells (HT-29).

## 2. Results

### 2.1. Bioactive Compounds Content and In Vitro Antioxidant Activities

The total content of bioactive compounds and in vitro antioxidant activities determined in the fermented (FS) and non-fermented spelt (NFS) flours are summarized in Table 1.

As shown in Table 1, bioactive compounds were significantly raised upon sourdough fermentation (*p* < 0.001 vs. NFS). Indeed, TPC and FC were increased by about 3.7- and 23-fold, respectively, in the fermented spelt flour to the non-fermented one.

As for in vitro antioxidant activity, several tests have been used, such as DPPH, FRAP, ORAC, and Fe^2+^ chelating ability. Likewise, for bioactive compounds, sourdough fermentation significantly increased from 2.7- to 7.3-fold the antioxidant properties of spelt (*p* < 0.01 vs. NFS). Specifically, the fermented spelt sample exhibited a considerably lower EC_50_ value for DPPH (7.3-fold) and iron chelating ability (3.7-fold) and higher FRAP (5.8-fold) and ORAC (2.7-fold) values than the non-fermented one.

### 2.2. Quantification of Phenolic Compounds by HPLC-DAD

The determination of FS and NFS flours’ phenolic compounds, performed by HPLC-DAD, is shown in Table 2. Gallic acid (49.71 mg/kg dw) was identified as the dominant component of fermented spelt flour, followed by rutin, trans-ferulic acid, iso-quercitrin, and quercetin. Instead, rutin and gallic acid (14.6 and 5.19 mg/kg dw, respectively) were determined as the dominant active component of the non-fermented spelt, while no other phenolic compounds were detected. Finally, 4-OH Benzoic acid, vanillic acid, vitexin, and trans-p-coumaric acid were detected neither in FS nor NFS.

In line with the spectrophotometric results, the HPLC-DAD analysis highlighted raised amounts of all detected phenolic compounds, excluding rutin, following sourdough fermentation and the concentrations of quercetin, iso-quercitrin, trans-ferulic acid, and gallic acid determined in FS flour were significantly increased from 2.3- to 9.6-fold than the NFS one (*p* < 0.05 vs. NFS).

### 2.3. Cellular Antioxidant Activity (CAA) and Hemolysis Assays in Red Blood Cells

**Figure 1:**
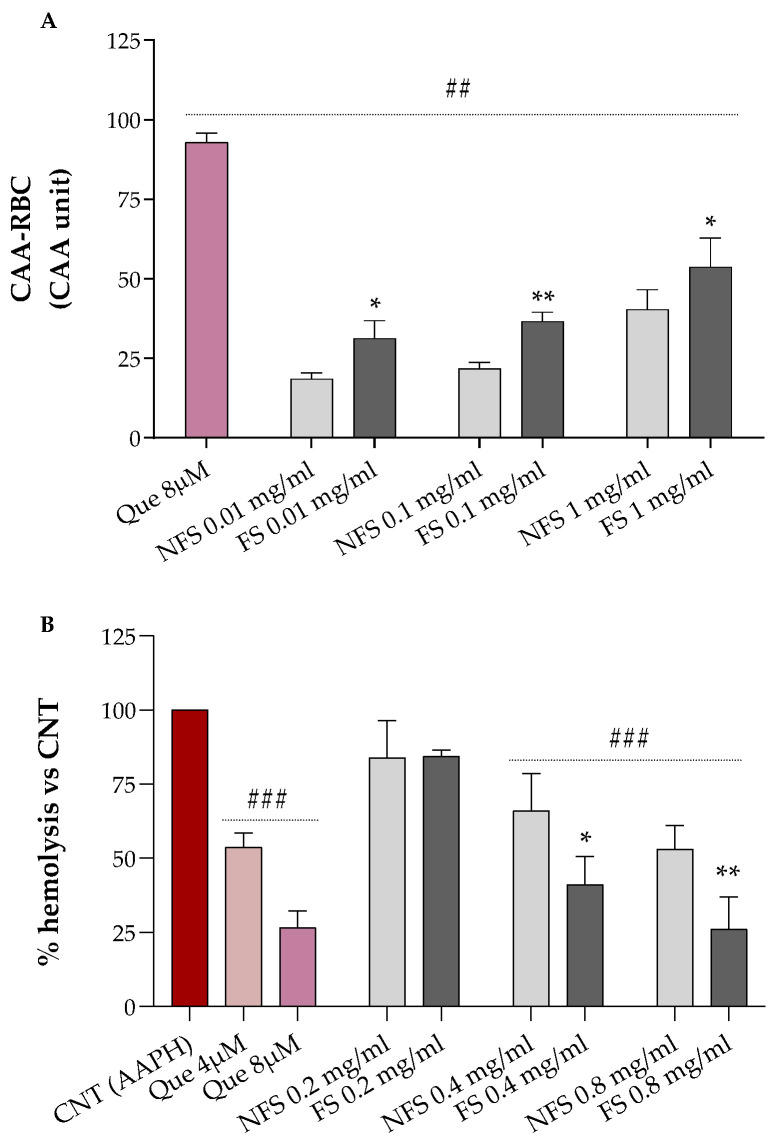
Protective effects of fermented (FS) and non-fermented spelt (NFS) flour extract, at different concentrations, on the cellular antioxidant activity—CAA (**A**) and % hemolysis (**B**) of human erythrocytes under oxidative conditions. Quercetin was used as a standard. Data were expressed as mean ± SD. One-way ANOVA with Bonferroni’s Multiple Comparison test: # significantly different from control, AAPH-treated cells (CNT, CAA = 0 and 100% hemolysis), ## *p* < 0.01, ### *p* < 0.001. Unpaired Student *t*-test: * significantly different from NFS, * *p* < 0.05, ** *p* < 0.01.

Results relative to the erythrocytes’ protection of increasing concentrations of FS and NFS flour extract against AAPH-induced oxidative stress are shown in Figure 1A (CAA-RBC) and Figure 1B (hemolysis test).

Both FS and NFS pre-treatments, at all tested doses (0.01, 0.1, and 1 mg mL^−1^), significantly raised the antioxidant activity of human erythrocytes by about 18–53% compared to the control (CAA = 0, *p* < 0.01 vs. CNT), with CAA values lower than the quercetin 8 µM (~93%), corresponding to 2.4 µg mL^−1^, which is about 4.17- to 416.7-fold lower than the concentrations used for our samples (Figure 1A). Additionally, all treatments enhanced the antioxidant properties of erythrocytes in a dose-dependent manner. At the same time, fermented spelt extract at all tested doses protected RBC against oxidative injury more than the non-fermented one (*p* < 0.05 vs. NFS).

**Figure 2:**
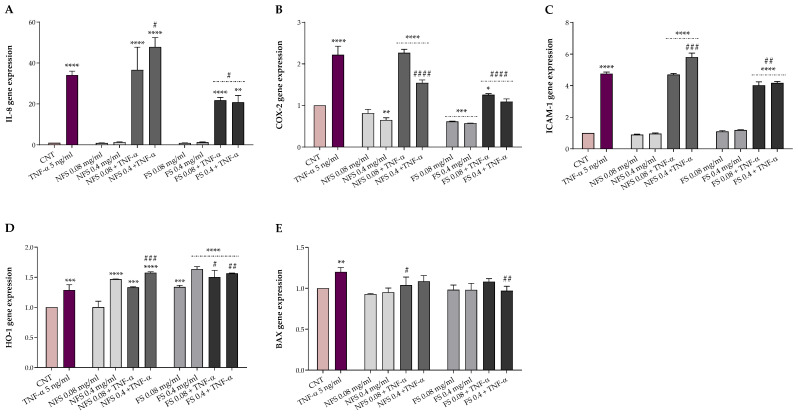
Quantitative Real-Time RT-PCR analysis of IL-8 (**A**), COX-2 (**B**), ICAM-1 (**C**), HO-1 (**D**), and BAX (**E**) gene expression in HT-29 1-h pre-treated with or without 0.08 or 0.4 mg mL^−1^ fermented (FS) and non-fermented (NFS) spelt extract, then exposed for 24 h to 5 ng mL^−1^ TNF-α. Experiments were carried out in triplicate, and results were expressed as gene expression fold increase with respect to control. One-way ANOVA with Dunnett’s multiple comparison test: * significantly different from control (CNT), * *p* < 0.05, ** *p* < 0.01, *** *p* < 0.001, **** *p* < 0.001; # significantly different from TNF-α, # *p* < 0.05, ## *p* < 0.01, ### *p* < 0.001, #### *p* < 0.0001.

As shown in Figure 2B, we can observe that FS and NFS, at the highest doses (0.4 and 0.8 mg mL^−1^), significantly protect erythrocytes from oxidative hemolysis compared to control cells exposed to the oxidizing agent alone (100% hemolysis, *p* < 0.001 vs. CNT). Moreover, both FS and NFS exerted a dose-dependent hemolysis inhibition with lysis protection values at 0.4 and 0.8 mg mL^−1^ comparable to the quercetin 4 µM or 8 µM, depending on the tested dose.

### 2.4. Angiotensin-Converting Enzyme (ACE) Inhibitory Activity of FS and NFS Peptide Extracts

The antihypertensive properties of FS and NFS peptide extracts were evaluated through the ACE-inhibitory test. As a result, peptides extracted from FS exhibited a significantly higher ACE inhibition than the NFS ones (*p* < 0.01 vs. NFS) with % inhibition values of 26.71 vs. 13.82, respectively. Therefore, the fermentation process increased the ACE inhibitory activity of spelt flour twice.

### 2.5. FS and NFS Protective Effects against TNFα-Induced Intestinal Alterations

The potential protective effects of FS and NFS against TNF-α-induced alterations were evaluated in HT-29, a human colonic adenocarcinoma cell line, exposed for 24 h to 5 ng mL^−1^ TNF-α following a 1-h pre-treatment with or without 0.08 and 0.4 mg mL^−1^ FS and NFS extracts. Preliminarily, cell viability was evaluated by MTT assay to exclude possible cytotoxic effects. The protective effects of both preparations were then explored, both at the basal condition and under TNF-α inflammatory insult, as gene expression of markers involved in inflammation (IL-8, COX-2, and ICAM-1), oxidative stress (HO-1), and apoptosis (BAX) (Figure 2).

As shown in Figure 2, FS and NFS pre-treatment did not impact the gene expression of IL-8, ICAM-1, and BAX at the basal condition. Otherwise, the pre-treatment with FS, at both concentrations, significantly decreased the COX-2 expression levels (*p* < 0.001 vs. CNT) while increasing the HO-1 ones (*p* < 0.001 vs. CNT). Conversely, with respect to the control, only the highest dose of NFS was able to reduce the basal expression of COX-2 (*p* < 0.01) and raise the HO-1 gene expression (*p* < 0.0001).

Instead, TNF-α inflamed HT-29 cells showed a significantly higher expression of all genes involved in inflammation (*p* < 0.001), oxidative stress (*p* < 0.001), and apoptosis (*p* < 0.01) compared to the control. However, the FS pre-treatment, at both concentrations, was able to counteract the alterations induced by TNF-α exposure, significantly reducing the IL-8 (*p* < 0.5), COX-2 (*p* < 0.0001), and ICAM-1 (*p* < 0.01) expression levels while increasing the HO-1 ones (*p* < 0.5). Moreover, FS extract, at the highest dose, lowered the BAX and COX-2 gene expression to the basal level. On the contrary, no modulatory effects on the gene expression of BAX were observed when HT-29 cells were pre-treated with the lowest dose of FS extract and exposed to the inflammatory insult. Regarding the non-fermented sample, NFS pre-treatment did not affect, at the lowest dose, the expression levels of IL-8, COX-2, ICAM-1, and HO-1 up-regulated by TNF-α exposure while reducing BAX levels (*p* < 0.05). In contrast, compared to TNF-α treated cells, the highest dose of NFS extract significantly reduced COX-2 (*p* < 0.0001) and increased HO-1 (*p* < 0.001) and, unexpectedly, the IL-8 (*p* < 0.05) and ICAM-1 (*p* < 0.001) levels. Finally, no variation in the BAX gene expression levels was found when cells were pre-treated with 0.4 mg mL^−1^ of NFS extract and exposed to TNF-α.

## 3. Discussion

In recent years, there has been growing interest in fermented and ancient cereal-based foods because of their supposed health benefits effects. Indeed, the fermentation process applied to cereals usually increases the bioactive compound content and the functional properties of derived products [4].

Our study focused on the impact of sourdough fermentation on the bioactive content and composition, as well as the antioxidant, antihypertensive, and anti-inflammatory properties of flour obtained from *T. dicoccum* (spelt) from Garfagnana (Lucca, Tuscany, Italy). This ancient cereal is gaining great attention for its supposed health-promoting effects and product sustainability. Our results showed that sourdough fermentation highly increased the spelt flour’s total phenolic and flavonoid content and its bio-functional properties. Indeed, following fermentation, we observed variation in the phenolic profile and detected a 3.7 to 23-fold increase in total phenolic and flavonoid content, respectively, in the fermented spelt flour. The higher total phenolic and flavonoid content reported herein aligns with those previously described in other fermented cereal flours [18,19]. Additionally, our results are consistent with what was observed by Colosimo et al. [16], who observed a time-dependent increase of amino acids, organic acids, and aromatic compounds with potential antioxidant activity following the fermentation of spelt flour. Among these, the increment in the amino acid concentration, such as phenylalanine, a known precursor of plants’ phenolic compounds, through the proteolytic activity of sourdough microbiota, might contribute directly to the synthesis and rise of phenolic compound levels [20].

To the best of our knowledge, the phenolic composition and concentration in Tuscan fermented spelt flour have not been yet reported. The HPLC-DAD analysis revealed the presence of gallic acid as the most representative compound of fermented spelt flour, followed by rutin, trans-ferulic acid, iso-quercitrin, and quercetin. Similarly, gallic acid was identified as the dominant component of fermented wheat (*T. aestivum*) flour [18]. Instead, rutin, followed by gallic acid, has been identified as the predominant polyphenol of non-fermented spelt flour. These findings disagree with previous results reported in the literature [21,22,23], in which ferulic acid was identified as the dominant phenolic component of *T. dicoccum*. However, according to the literature, changes occurring in the bioactive compound content and composition of fermented spelt flour may be due to the microorganisms’ hydrolytic activities that, via glycosides, acyl glycosides, or polymers hydrolysis, can release more and/or new compounds [24].

In line with the rise in the phenolic and flavonoid content, a significant increase in the in vitro antioxidant activities was found following the spelt flour’s sourdough fermentation (Table 1). Our results align with what was observed by Colosimo et al. [16], who described a time-dependent rise in the antiradical ability of fermented spelt flour after sourdough fermentation. Additionally, the higher antioxidant activities detected in fermented spelt flour are consistent with those previously observed in different fermented cereals, including wheat, millet, maize, sorghum, oats, etc. [4]. Our findings are in line with what was reviewed by Hur et al. [25] and Đorđević et al. [26], who highlighted the ability of fermentation to improve the antioxidant activity of several plant materials through the increase of phenolic and flavonoid compounds concentration as a result of a microbial hydrolysis reaction. Moreover, as reviewed by Gabriele and Pucci [4], the increase in free and soluble phenols concentration depends on both cereals substrate and fermenting conditions, besides microorganisms involved in the release of bound phenols. Nonetheless, during fermentation, other metabolic processes/mechanisms could underlie the increase in phenolic compounds, and the increased antioxidant activity could be mediated by the aromatic amino acid residues’ protons donation to radicals that lack electrons [16]. Polyphenols exhibit their protective function through three main mechanisms, including hydrogen atom and/or electron transfer to free radicals and transition metal ions chelation (e.g., iron (II), copper (I)), which are involved in the free radicals generating process [27]. Therefore, more than one in vitro antioxidant method is necessary to cover the above anti-radical mechanisms. Among these, ORAC, FRAP, and Fe^2+^ chelating assays were respectively chosen for hydrogen atom transfer, single electron transfer, and transition metals chelation mechanisms, whereas the DPPH assay was used for its mixed hydrogen atom and electron transfer antiradical mechanism [28].

However, antioxidant in vitro assays do not often reflect the biological property of analyzed products, and RBC-based tests were, thus, used to provide complementary information. As shown in Figure 1, the fermented spelt flour exhibited better antioxidant properties than the non-fermented one and strongly protected RBC against oxidative AAPH-induced hemolysis. Our findings agree with those obtained using 72–96 h fermented spelt flour [16]. Similar results were also reported for sourdough-fermented wheat flour [18]. The improvement of the antioxidant properties of fermented *T. dicoccum* may be related to the increment of concentration and changes in the polyphenols composition, and the increase in the concentration of polyphenols, free amino acids, peptides, and other bioactive compounds may be attributed to this increment [17].

ACE is a protease involved in the control of blood pressure. Its increment beyond the normal causes an overproduction of angiotensin II, the main contributor to hypertension [14]. Recent studies have focused on food proteins as an alternative for hypertension prevention and treatment because they are free from side effects compared to current drugs such as enalapril, ramipril, and captopril [14]. Food proteins contain bioactive peptides that may be released via different ways, such as microbial fermentation, in vitro enzymatic hydrolysis, and gastrointestinal digestion. Once released, they could exert many functions, including antioxidant and ACE-inhibitory activity [29].

Our results showed that the antihypertensive property of spelt flour, assessed as ACE-inhibitory activity, highly increased upon sourdough fermentation. The antihypertensive properties of fermented and non-fermented spelt flours have not yet been reported, but similar results have been observed previously in other cereals. For instance, Ayyash et al. [30] found a several-fold increase in the ACE-inhibitory activity of wheat and quinoa after solid-state fermentation using *Lactobacillus* spp., while Wu et al. [31] reported a high ACE-inhibitory activity in oats fermented by *Rhizopus oryzae* and co-inoculation of the latter with *Lactobacillus plantarum* compared to the non-fermented ones. Instead, Wronkowska et al. [32] did not observe any changes in the ACE-inhibitory activity following buckwheat flour fermentation. Based on previous data available in the literature, the increment in ACE-inhibitory activity may be due to bioactive peptides released during fermentation via the protease activities of microorganisms. In addition, the fermentation condition and the plant material could contribute positively and differently to the release of ACE-inhibitory peptides.

Inflammation is a physiological response of the body to disease, infection, and injury characterized by the modulation of several inflammatory mediators via different pathways and mechanisms. However, a non-controlled and inadequate activation may lead to chronic inflammation [33] with negative health implications. Nowadays, thanks to their limited side effects and anti-inflammatory properties, natural products and plant-derived compounds represent promising tools for the management and/or treatment of intestinal inflammation [12,13]. Moreover, many studies have well-documented the role of cereal and cereal-based fermented products in inflammatory responses [34,35]. In this study, the anti-inflammatory properties of 1-h pre-treatment with fermented and non-fermented *T. dicoccum* flour extracts (0.08 and 0.04 mg mL^−1^) were assessed through the HT-29 model following a 24 h exposure to TNF-α (5 ng mL^−1^), used as an inflammatory stimulus, by evaluating the expression of genes involved in inflammation (IL-8, COX-2, and ICAM-1), oxidative stress (HO-1), and apoptosis (BAX). As previously reported, we used the lowest concentration of TNF-α, without toxicity, able to induce inflammation [18]. Our findings showed that both fermented and non-fermented pre-treatment did not impact the expression of pro-inflammatory genes at the basal condition. Instead, fermented extracts highly reduced the expression of inflammatory mediators counteracting the TNF-α-intestinal induced alterations. These results agree with those observed in TNF-α-inflamed HT-29 when pre-treated with fermented wheat extracts [18] and those obtained in LPS-stimulated HT-29 using bound polyphenols from foxtail millet bran [36]. The inhibition of IL-8, COX-2, and ICAM-1 herein detected suggests that fermented spelt flour may be useful in the treatment and/or management of intestinal inflammation.

In addition, the pre-treatment with both doses of the fermented spelt extract and the highest of the non-fermented one strongly increased the expression of HO-1 at both basal and inflammatory conditions. These findings are in accordance with those obtained in TNF-α-stimulated HT-29 using fermented wheat flour [18] and align with the higher antioxidant activity and bioactive compounds content detected following spelt flour fermentation. Among others, phenolic compounds appear to exhibit their antioxidant protective function via the activation of the nuclear factor (erythroid-derived 2)-like 2 (Nrf2), a transcription factor that regulates the expression of genes involved in oxidative stress, and the inhibition of nuclear factor-κB (NF-kB) that mediates inflammatory processes. Functional crosstalk between Nrf2 and NF-κB signaling pathways is critical for maintaining a balanced inflammatory response [4], and some evidence showed that the antioxidant enzyme HO-1, a target gene of Nrf2, plays a crucial role in cell protection thanks to its antioxidant and anti-inflammatory properties [37,38]. These results suggest that fermented spelt flour may mitigate inflammation reactions while strengthening the antioxidant enzyme system with potential therapeutic applications in inflammatory intestinal disease treatment. Finally, although we observed no changes in BAX expression at the basal condition, both fermented and non-fermented extracts significantly lowered the BAX expression under the inflammatory stimulus.

## 4. Materials and Methods

### 4.1. Chemicals and Reagents

Folin-Ciocalteu reagent, sodium carbonate, ethylenediaminetetraacetic acid (EDTA), sodium acetate, potassium chloride, sodium hydroxide, gallic acid, quercetin dihydrate, catechin hydrate, phosphate buffer saline (PBS) tablets, 6-hydroxy-2,5,7,8-tetramethylchromane-2-carboxylic acid (Trolox), 2,2-diphenyl-1-picrylhydrazyl (DPPH), 2,4,6-Tri(2-pyridyl)-s-triazine (TPTZ), ferric chloride hexahydrate (FeCl_3_·6H_2_O), ferrous sulfate heptahydrate (FeSO_4_·7H_2_O), ferrozine, sodium nitrite (NaNO_2_), aluminum chloride (AlCl_3_), 2,2-azobis (2-amidinopropane) dihydrochloride (AAPH), fluorescein sodium salt, 2′-7′dichlorofluorescein diacetate (DCFH-DA), dimethyl sulfoxide (DMSO), medium, and supplements for cell culture were purchased from Fluka-Sigma-Aldrich, Inc. (Saint Louis, MO, USA). Ethanol and methanol were purchased from VWR (Radnor, PA, USA), while hydrochloric acid was purchased from Merck (Readington, NJ, USA). All HPLC standards, including gallic acid, 4-OH benzoic acid, vanillic acid, rutin, vitexin, iso-quercitrin, trans-p-coumaric acid, trans-ferulic acid, and quercetin, as well as methanol (HPLC grade), acetonitrile (gradient HPLC grade), and phosphoric acid (ACS grade) solvents, were purchased from Sigma-Aldrich (Sigma Aldrich Chemie GmbH, Steinheim, Germany). Double deionized water (ddH_2_O) was treated (18.2 MΩ/cm, 20 °C) in a Simplicity 185 purification system (Millipore SAS, Molsheim, France). All reagents, media, and medium supplements for cell culture were purchased from Sigma-Aldrich (St. Louis, MO, USA).

### 4.2. Plant Material and Extraction

*Triticum dicoccum* (spelt) was supplied by “Unione dei Comuni della Garfagnana” (Lucca, Italy). The fermented sample was prepared from grounded spelt flour mixed with water containing a Cl^−^ concentration minor or equal to 0.8 mg/L to avoid interfering with the fermentation process due to its antimicrobial activity. The hydrated flour was then mixed with sourdough, provided by “Lievitamente s.n.c.” (Viareggio, Italy), in a steel container for food adapted to guarantee a continuous flow of air and a constant temperature of 38 °C under pH monitoring [16]. The fermentation was performed with continuous mixing for 96 h. Fermented and non-fermented spelt flours (80 mg mL^−1^) were sonicated (three cycles: 10 s on/10 s off) and shaken gently for 1 h while being extracted with 10% DMSO in distilled water. Samples were centrifuged for 10 min at 2300× *g* at 4 °C (Jouan CR3i centrifuge, Newport Pagnell, UK), and the supernatant was collected, filtered (0.2 µm VWR International PBI, Milan, Italy), and kept at 4 °C in the dark until use. The extraction was carried out in triplicate.

### 4.3. Bioactive Compounds Content

The total phenolic and flavonoid content of fermented (FS) and non-fermented spelt (NFS) flours were estimated as previously described [39]. Total phenolic content, estimated as Folin-Ciocalteu (FC) reducing capacity, was expressed as mg of gallic acid equivalents (GAE)/g dry weight (DW), and the absorbance was read at 760 nm (Perkin Elmer UV/VIS Lambda 365, Waltham, MA, USA). Flavonoids, quantified using the aluminum chloride colorimetric method, were expressed as mg quercetin equivalent (QE)/g FW, and the absorbance was recorded at 430 nm.

### 4.4. In Vitro Antioxidant Activities

The DPPH• radical scavenging activity of FS and NFS extracts was determined according to Chiellini et al. [40]. The absorbance was recorded at 517 nm, and the extract concentration corresponding to 50% of DPPH inhibition (EC_50_) was measured according to Gabriele et al. [41]. The oxygen radical absorbance capacity (ORAC) of FS and NFS extracts was determined as described by Gabriele et al. [39]. AAPH was used as a peroxyl radicals generator and fluorescein as the probe. Fluorescein fluorescence decay was read at λ_ex_ 485 nm and λ_em_ 514 nm using a VictorTM X3 Multilabel Plate Reader (Perkin Elmer, Waltham, MA, USA). Results were expressed as ORAC units (µmol TE/g FW) using Trolox as the reference standard. The ferric-reducing antioxidant power (FRAP) assay was used to evaluate the ability of FS and NFS extracts to reduce ferric iron (Fe^3+^) to ferrous iron (Fe^2+^) [42]. The absorbance was measured at 593 nm (Perkin Elmer UV/VIS Lambda 365, Waltham, MA, USA), and results were expressed as Fe^2+^ equivalents (µM) using a standard curve of FeSO_4_·7H_2_O. The Fe^2+^ chelation ability of FS and NFS extracts was determined as described by Chelucci et al. [42]. The absorbance was read at 562 nm (Perkin Elmer UV/VIS Lambda 365, Waltham, MA, USA), and results were expressed as EC_50_ values referring to the extract concentration corresponding to 50% of Fe^2+^ chelation.

### 4.5. Polyphenols Quantification by HPLC-DAD

The FS and NFS samples were extracted with 80% methanol (*v*/*v*) at laboratory temperature for 2 h by horizontal shaker Unimax 2010 (Heidolph Instruments GmbH, Schwabach, Germany). The extract was filtered through Munktell No 390 paper (Munktell & Filtrak GmbH, Bärenstein, Germany) and stored in closed 20 mL PE vial tubes. Prior to HPLC analysis, the extract was filtered through syringe filter Q-Max (0.22 µm, 25 mm, PVDF) (Frisenette ApS, Knebel, Denmark). All compounds were determined using an Agilent 1260 Infinity HPLC (Agilent Technologies GmbH, Wäldbronn, Germany) with quaternary solvent manager coupled with degasser (G1311B), sampler manager (G1329B), column manager (G1316A), and DAD detector (G1315C). All HPLC analyses were performed on a Purosphere^®^ reverse phase C18 column (250 mm × 4 mm × 5 µm) (Merck KGaA, Darmstadt, Germany). The mobile phase consisted of gradient acetonitrile (A) and 0.1% phosphoric acid in ddH_2_O (B). The gradient elution was as follows: 0–1 min isocratic elution (20% A and 80% B), 1–5 min linear gradient elution (25% A and 75% B), 5–15 min (30% A and 70% B), and 15–25 min (40% A and 60% B). The post-run was 3 min. The initial flow rate was 1 mL/min, and the injection volume was 5 μL. The column thermostat was set up to 30 °C, and the samples were kept at 4 °C in the sampler manager. The detection wavelengths were set up at 265 nm (4-OH benzoic acid, vanillic acid, rutin), 320 nm (gallic acid, vitexin, trans-p-coumaric acid, trans-ferulic acid), and 372 nm (iso-quercitrin, quercetin). Data were collected and processed using Agilent Open Lab Chem Station software version c 01.06 for LC 3D systems.

### 4.6. Cellular Antioxidant Activity (CAA) and Hemolysis Assays

Human blood samples were collected in ethylenediaminetetraacetic acid (EDTA)-treated tubes and centrifuged for 10 min at 2300× *g* at 4 °C. Plasma and buffy coat were discarded, and erythrocytes were washed twice with PBS pH 7.4. The cellular antioxidant activity of FS and NFS extracts was tested on human erythrocytes as previously described [43]. Quercetin was used as the reference standard. Results were expressed as CAA units using the following formula: 100 − (∫SA/∫CA) × 100, where ∫SA is the integrated area of the sample curve and ∫CA is the integrated area of the control curve.

The anti-hemolytic effects of FS and NFS extracts were evaluated on oxidized human erythrocytes as described by Frassinetti et al. [43]. Quercetin was used as the reference standard. Results were expressed as a percentage of hemolysis with respect to control, referring to AAPH-treated erythrocytes.

### 4.7. Peptide Extraction and Angiotensin-Converting Enzyme (ACE) Inhibitory Activity

Peptide extraction was carried out as described by Vilcacundo et al. [44]. Briefly, FS and NFS flours were mixed 1:10 (*w*/*v*) with distilled water, shaken for 1 h, and centrifuged, after adjusting the pH to 12 with NaOH (1 M), at 4500× *g* at 25 °C for 30 min. Supernatants were collected and centrifugated at 4500× *g* at 4 °C for 20 min after adjusting the pH to 4.0 with HCl (2 M). The pellets were dissolved in water, neutralized with NaOH (0.1 M), and lyophilized before storing at −20 °C until use. The protein content was measured using the method of Lowry [45].

The ACE-inhibitory activity of lyophilized FS and NFS peptide extracts was determined according to Arouna et al. [46]. Enalapril was used as the positive control, while bidistilled water was used as the negative one. The percentage of ACE inhibition was calculated as follows: % ACE inhibition = (A_negative_ − A_sample_)/A_negative_ × 100, where A_negative_ and A_sample_ are the absorbances of negative and diluted samples, respectively.

### 4.8. Human Colonic Cell Line Treatments and Viability

The human colonic adenocarcinoma cell (HT-29) line (DSMZ, Braunschweig, Germany) was grown as previously reported [18]. All treatments were carried out using DMEM + F12 medium without phenol red and FBS, containing antibiotics. HT-29 cells were pre-treated for 1 h with or without FS and NFS extracts (0.08 and 0.4 mg mL^−1^), then exposed for 24 h with or without 5 ng mL^−1^ TNF-α. HT-29 cell viability was evaluated by the MTT assay as previously described [47].

### 4.9. RNA Extraction, RT-PCR, and Quantitative Real-Time RT-PCR

Total RNA was isolated from HT-29 cells using the E.Z.N.A.^®^ Total RNA Kit I (OMEGA Bio-Tek, Norcross, GA, USA) and reverse-transcribed using the iScript™ cDNA Synthesis Kit (Bio-Rad, Hercules, CA, USA). Quantitative Real-Time PCR was performed using the SsoFast™ EvaGreen^®^ Supermix (Bio-Rad, Hercules, CA, USA) in a CFX Connect Real-Time PCR Detection System (Bio-Rad, Hercules, CA, USA). IL-8 (C-X-C motif chemokine ligand 8), COX-2 (prostaglandin-endoperoxide synthase 2), ICAM-1 (intercellular adhesion molecule-1), BAX (BCL2 associated X, apoptosis regulator), HO-1 (heme oxygenase-1), and β-actin gene primers were described previously [18]. The gene expression was calculated by the 2^−ΔΔCT^ relative quantification method.

### 4.10. Statistical Analysis

Statistical analysis was performed using GraphPad Prism, version 6.00 for Windows (GraphPad Software, San Diego, CA, USA). Assays were carried out in triplicate, and results were expressed as mean values ± standard deviation (SD). Differences between samples were analyzed by one-way analysis of variance (ANOVA) with Dunnett or Bonferroni multiple comparison tests. Unpaired Student’s *t*-test was used for fermented and non-fermented spelt flour comparison. A *p*-value lower than 0.05 was considered statistically significant.

## 5. Conclusions

The present study analyzed the impact of sourdough fermentation on the content and composition of bioactive compounds of *T. dicoccum* (spelt) flour and its antioxidant, antihypertensive, and anti-inflammatory properties. Evidence from this study suggests moderate antioxidant, antihypertensive, and anti-inflammatory properties of the non-fermented spelt sample. Instead, sourdough fermentation increased the total phenolic and flavonoid compound content, modified the phenolic composition, and improved the spelt flour’s antioxidant and anti-inflammatory properties. The antihypertensive effect of non-fermented spelt flour suggests the pre-existence of ACE-inhibitory compounds in the raw material; instead, the higher inhibition activity of fermented sample may be due to the synergistic effects between bioactive components released following fermentation and the pre-existing compounds that have not undergone any changes during fermentation. Sourdough fermentation positively affected the nutraceutical and functional properties of spelt that may mitigate inflammation reactions while strengthening the antioxidant system, representing a valuable ingredient for the formulation of functional foods with potential therapeutic applications in the management and/or treatment of hypertension and oxidative and inflammatory intestinal diseases.

## Figures and Tables

**Table 1:** Bioactive compounds content (total phenolic and flavonoid content) and in vitro antioxidant activities (DPPH, FRAP, ORAC, and Fe^2+^ chelating ability) of fermented (FS) vs. non-fermented spelt (NFS) flour. Results were expressed as mean values ± SD of three replicates. Unpaired Student *t*-test: * significantly different from NFS: ** *p* < 0.01, *** *p* < 0.001.

	TPC (mg GAE/g dw)	FC (mg CE/g dw)	DPPH EC_50_ (mg mL^−1^)	FRAP (µM Fe^2+^)	ORAC (μmol TE/100 g dw)	Fe^2+^ Chelation EC_50_ (mg mL^−1^)
NFS	0.99 ± 0.08	0.23 ± 0.02	15.64 ± 1.90	268.53 ± 26.74	212.66 ± 71.85	18.79 ± 1.70
FS	3.65 ± 0.17 ***	5.33 ± 0.11 ***	2.15 ± 0.51 ***	1565.33 ± 111.09 ***	577.09 ± 96.18 **	5.07 ± 0.67 ***

* TPC: total phenolic content; FC: flavonoid content; DPPH: 2,2-diphenyl-1-picrylhydrazyl; FRAP: Ferric Reducing Antioxidant Power; ORAC: Oxygen Radical Absorbance Capacity; GAE: gallic acid equivalent; CE: catechin equivalent; TE: trolox equivalent; dw: dry weight; EC_50_: half maximal effective concentration.

**Table 2:** Phenolic compounds, class, retention time (Rt, min), wavelengths (nm), and concentrations (mg/kg dw) in the fermented (FS) and non-fermented (NFS) spelt flour. Unpaired Student *t*-test: * significantly different from NFS: * *p* < 0.05, ** *p* < 0.01, *** *p* < 0.001.

Phenolic Compound	Rt	Wavelength	Concentration		Class
			FS	NFS	
Gallic acid	2.669	320	49.71 ± 5.28 **	5.19 ± 0.14	Phenolic acid
4-OH Benzoic acid	4.830	265	≤LOD	≤LOD	Carboxylic acid
Vanillic acid	5.260	265	≤LOD	≤LOD	Phenolic acid
Rutin	5.955	265	10.5 ± 1.14 *	14.6 ± 0.32	Flavonol
Vitexin	6.275	320	≤LOD	≤LOD	Flavone
iso-quercitrin	6.987	372	2.61 ± 0.08 ***	≤LOD	Flavonol
trans-p-Coumaric acid	7.444	320	≤LOD	≤LOD	Hydroxycinnamic acid
trans-Ferulic acid	8.219	320	8.74 ± 0.82 **	≤LOD	Hydroxycinnamic acid
Quercetin	17.363	372	2.25 ± 1.13 *	≤LOD	Flavonol

LOD: limit of detection; dw: dry weight.

## Data Availability

All data is contained within the article.
